# The status of RpoS impacts the 2-aminoacrylate stress paradigm in *ridA* mutants of *Salmonella enterica* serovar Typhimurium

**DOI:** 10.1128/jb.00390-26

**Published:** 2026-09-18

**Authors:** Andrea Giuliano, Jessica K. Novak, Diana M. Downs

**Affiliations:** 1 Department of Microbiology, University of Georgia 189270, Athens, Georgia, USA; University of Florida, Gainesville, Florida, USA

**Keywords:** RpoS, RidA, 2-aminoacrylate, metabolic stress

## Abstract

Bacterial metabolism is composed of a complex system of anabolic and catabolic processes, and productive growth results from the regulated interplay of these processes. Genetic and biochemical studies on the LT2 type strain of *Salmonella enterica* serovar Typhimurium determined the need for RidA to prevent metabolic stress caused by 2-aminoacrylate (2AA). RidA is an enamine deaminase, and in its absence, 2AA accumulates and can irreversibly damage critical pyridoxal 5′-phosphate-dependent enzymes. Unexpectedly, the disruption of *ridA* in a virulent type strain (*S. enterica* serovar Typhimurium strain 14028s) did not replicate the serine sensitivity observed in LT2. Consistent with reported differences between these strains, the status of *rpoS* impacted the phenotypes caused by a *ridA* mutation. Specifically, serine sensitivity in the 14028s strain background occurred only when an insertion in *rpoS* was combined with a mutation in *ridA*. Further data suggest the 14028s *rpoS ridA* growth deficiency is due to poor expression of *sdaA*, which encodes an Fe-S cluster serine deaminase. Additional combinatorial effects of lesions in *ridA* and *rpoS* indicate that the functional status of RpoS modulates the metabolic network in multiple ways that impact the cellular response to 2AA.

**IMPORTANCE:**

RidA is an enamine/imine deaminase known for mediating 2-aminoacrylate (2AA)-induced stress in several bacterial models. Best characterized in *S. enterica*, manipulations to RidA have been used to probe metabolic flux in various bacteria. Here, we find that a key factor in the manifestation of 2AA stress is the status of transcriptional regulator RpoS. Our results indicate that a functional RpoS mediates some of the stress induced by exogenous serine in a *ridA* mutant strain and further implicates this regulator in modulating the metabolic network. These results bolster our understanding of the flux and robustness integral to bacterial metabolism and emphasize a need to better understand the involvement of the RpoS regulon.

## INTRODUCTION

**Fig 1:**
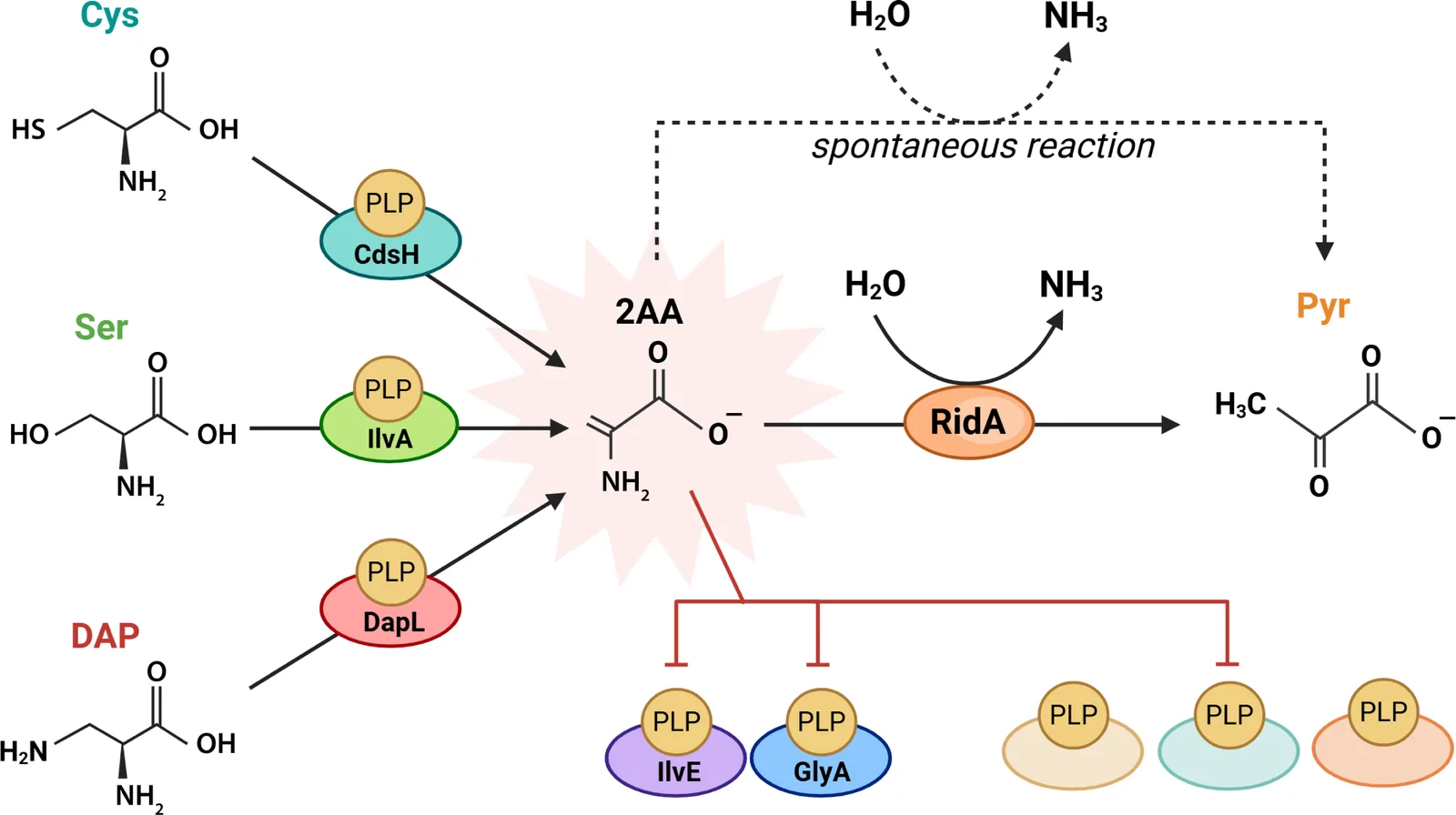
RidA prevents the accumulation of 2-aminoacrylate (2AA). The paradigm for 2AA stress as defined in *S. enterica* is schematically shown. Some pyridoxal 5′-phosphate (PLP)-dependent enzymes (PLPDEs), including serine/threonine dehydratase IlvA, cysteine desulfhydrase CdsH, and diaminopropionate ammonia-lyase DapL, generate reactive 2AA enamine, which is released into the cellular milieu. 2AA is deaminated to generate the more stable ketoacid pyruvate. This reaction is catalyzed by RidA but can occur more slowly via spontaneous deamination. If it accumulates, 2AA can attack the PLP in cellular enzymes (including transaminase B IlvE and serine hydroxymethyltransferase GlyA) to generate a covalently modified, and thus inactivated, enzyme. In the absence of RidA, the reduction in enzymatic activities from accumulated 2AA can result in detectable growth defects. Created in BioRender. Novak, J. (2026) https://BioRender.com/kzc6kbg.

Microbes maintain a robust and resilient metabolism that ensures the ability to adapt and thrive in often changing environments. Maintaining a vibrant metabolic network involves not only the appropriate components but also a complex set of regulatory inputs that act on multiple levels through a variety of mechanisms. In total, cellular metabolism involves the integration of biochemical reactions and pathways, which is often mediated by chemistry and metabolites (1). Resilience of a metabolic network is facilitated by robustness, the capacity of a system to compensate for the loss or perturbation of one node by remodeling or utilizing another (2). The ability to maintain a functional system in the face of perturbation is a critical component of microbial metabolic networks (3). The complexities of metabolic network design and regulation can be effectively probed with a model system in which a minor perturbation can have a dramatic, measurable impact. Several such systems have been described in *S. enterica* and have been exploited to understand pathway connections and metabolic integration (4–7). The role of RidA in 2-aminoacrylate (2AA) stress represents one such system (Fig. 1) (8, 9).

RidA (**r**eactive **i**ntermediate **d**eaminase A) is a small protein conserved in all domains of life and the archetypical member of the large Rid protein family that has seven subfamilies (10). The RidA protein has been a central component of several studies addressing metabolic stress and consequences of perturbing the metabolic network (4, 11–13). 2AA is released as a reaction product from several pyridoxal 5′-phosphate (PLP)-dependent enzymes and accumulates in the absence of RidA. Accumulation of 2AA can damage PLP-dependent enzymes (14). Specifically, as a highly potent electrophile, 2AA spontaneously reacts with nucleophiles to form a covalent bond; the PLP cofactor is particularly susceptible. The reaction of a PLP-dependent enzyme with 2AA results in a covalent 2AA-derived modification that inactivates the enzyme. Since 2AA damage is post-translational and irreversible, it can be detected as a decrease in activity of the attacked enzymes (8, 15, 16). Transaminase B (IlvE, EC 2.6.1.27) is one target enzyme of 2AA and has been used as a proxy for 2AA accumulation (8, 17).

Despite the conserved enamine/imine deaminase activity of RidA homologs (18), the phenotypic impact of eliminating RidA differs between organisms (12, 19–24). These phenotypic differences appear to reflect distinct thresholds for enzymatic activity, metabolite pool sizes, and/or metabolic network configuration. Studies that characterize the 2AA/RidA stress paradigm in various bacterial hosts and other organisms can emphasize and elaborate on differences in metabolic network architecture.

Perturbation of the metabolic network can generate and/or be caused by various stressors, only one of which is 2AA. Microbes have invested significant resources to prevent and/or repair stressor-induced damage. The RpoS protein, which is found in most γ-, β-, and δ-proteobacteria (25), is involved in the response to a variety of stresses. RpoS is a group 2 sigma factor and the central regulator of the general stress response, directing core RNA polymerase to a specific set of promoters, collectively controlling numerous genes (~500 in *Escherichia coli*) (25–27). Stresses like nutrient deprivation, oxidative stress, changes in aerobic/energy status, stationary phase, and extreme pH promote the synthesis and stabilization of RpoS (28, 29). Stabilization of RpoS triggers a general stress response that activates pathways involved in toxin elimination, cellular protection, amino acid degradation, metabolic optimization, biofilm formation, and virulence, among others (25).

In *S. enterica*, RpoS regulates genes of the *spv* virulence plasmid and genes that enable survival under conditions likely encountered during intracellular growth within host macrophages (30). In addition, RpoS controls chromosomal genes that contribute to the colonization of Peyer’s patches and to the long-term persistence of infection in mice (31). Thus, it is not surprising that the virulent *S. enterica* Typhimurium 14028s strains differ from the attenuated laboratory LT2 strains at the *rpoS* locus (32). The *rpoS* gene in the LT2 reference genome contains the rare UUG start codon (33). Consequently, an LT2 strain translates lower levels of RpoS than a 14028s strain, which compromises its ability to respond to various stresses and ultimately impairs the capacity to colonize and persist in key host organs (33). Our understanding of the breadth and consequences of metabolic changes caused by the lack of RpoS is incomplete.

The current study was initiated to determine the metabolic basis for phenotypic differences between *ridA* mutants of strain LT2 and strain 14028s. The data show that a mutation of *rpoS* alters the consequences of a *ridA* mutation on the metabolic network and fitness of *S. enterica*. Specifically, RpoS relieves the serine sensitivity characteristic of a *ridA* (LT2) mutant. The data show that this effect is at least partially due to RpoS-dependent regulation of *sdaA*, which encodes the serine dehydratase SdaA (EC 4.3.1.17). Combinatorial effects of lesions in *ridA* and *rpoS* suggest that the functional status of RpoS modulates the metabolic network in several ways that impact the cellular response to 2AA.

## RESULTS

### Strains of *Salmonella enterica* differ in their response to a *ridA* mutation

**Fig 2:**
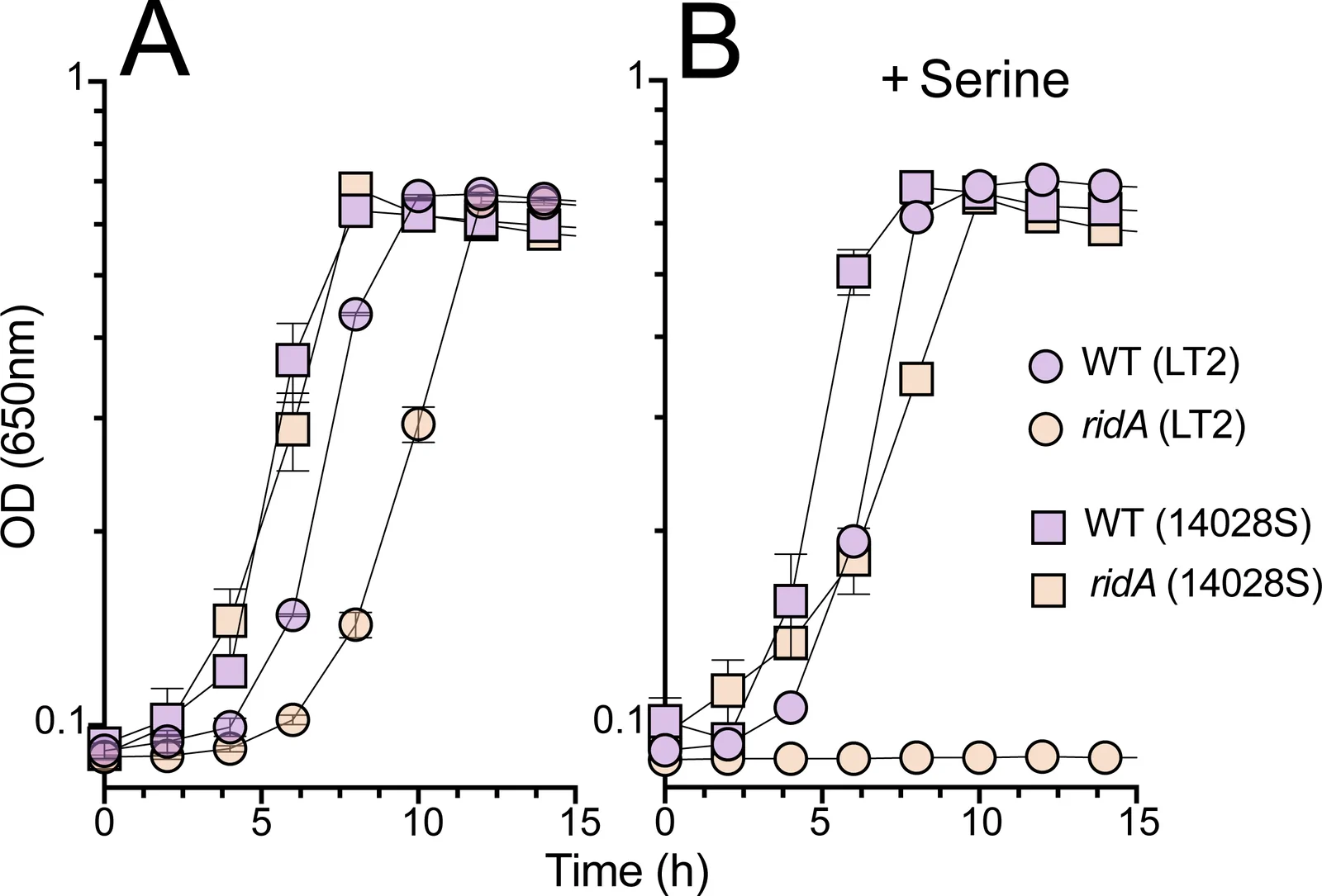
*ridA* mutants of *S. enterica* have different sensitivity to serine. Four strains: WT (LT2) (DM9404), WT (14028s) (DM18363), *ridA* (LT2) (DM3480), and *ridA* (14028s; DM18680) were grown at 37°C in glucose minimal medium containing no addition (**A**) or 5 mM L-serine (**B**). The data reflect experiments performed in biological triplicate, and error bars represent the standard deviation.

LT2 is the strain of *S. enterica* that has been used in metabolic and molecular biological studies of *Salmonella* over many decades (34–38). In contrast, strain 14028s is commonly used as the wild-type *S. enterica* strain in virulence studies (39–42). *ridA* mutants of LT2 (denoted *ridA* [LT2] throughout) accumulate 2AA, which causes multiple defects resulting from damaged PLP-dependent enzymes. The generation of, and reaction to, 2AA by *S. enterica* LT2 has been the basis for defining the paradigm of 2AA stress (9, 17, 23). An insertion in *ridA* was transduced by phage P22 into *S. enterica* strain 14028s, resulting in strain DM18680. Unexpectedly, DM18680 (*ridA* [14028s]) did not show sensitivity to serine (5 mM) in glucose minimal medium that is characteristic of *ridA* (LT2) mutants (Fig. 2). Titration confirmed that the *ridA* (LT2) mutant failed to grow in the presence of 0.625 mM serine, while growth of the *ridA* (14028s) mutant was not affected by serine concentrations of 5 mM (Fig. S1).

### Strains LT2 and 14028s carry different alleles of *rpoS*

Strain LT2 is reported to be avirulent due to a decreased function of RpoS resulting from an atypical start codon (30–33). Whole-genome sequencing (WGS) was performed on the LT2 and 14028s strains in our laboratory collection. WGS was done by SeqCenter Laboratory (Pittsburgh, Pennsylvania), and the resulting reads were processed in our laboratory using the Breseq open-source pipeline (43). Sequencing reads from LT2 were aligned to the *S. enterica* Typhimurium LT2 reference genome (GenBank AE006468.2), while reads from 14028s were aligned to the *S. enterica* Typhimurium 14028s reference genome (GenBank CP001363.1). Differences between the LT2 and 14028s laboratory strains were then determined by aligning reads from LT2 to the 14028s reference genome. Notably, there were multiple sequence differences between the LT2 and 14028s strains housed in our laboratory when compared to the reference genomes of each respective strain. Since the *rpoS* gene is involved in the virulence phenotypes of the two strains, we focused first on allelic differences in *rpoS*. As expected, the 14028s strain carried the canonical ATG start codon, in contrast with a TTG in the LT2 strain (32). Unexpectedly, the *rpoS* gene in the laboratory LT2 strain also carried an eight-base pair deletion from 3,066,142 to 3,066,149 ntbp. This mutation is localized to the 3′ end of the sequence and corresponds to the C-terminus of the protein that contains the DNA-binding domain. The eight-base pair deletion results in a frameshift, which likely aberrates this domain (Fig. S2).

### The status of *rpoS* contributes to serine sensitivity of a *ridA* mutant

**Fig 3:**
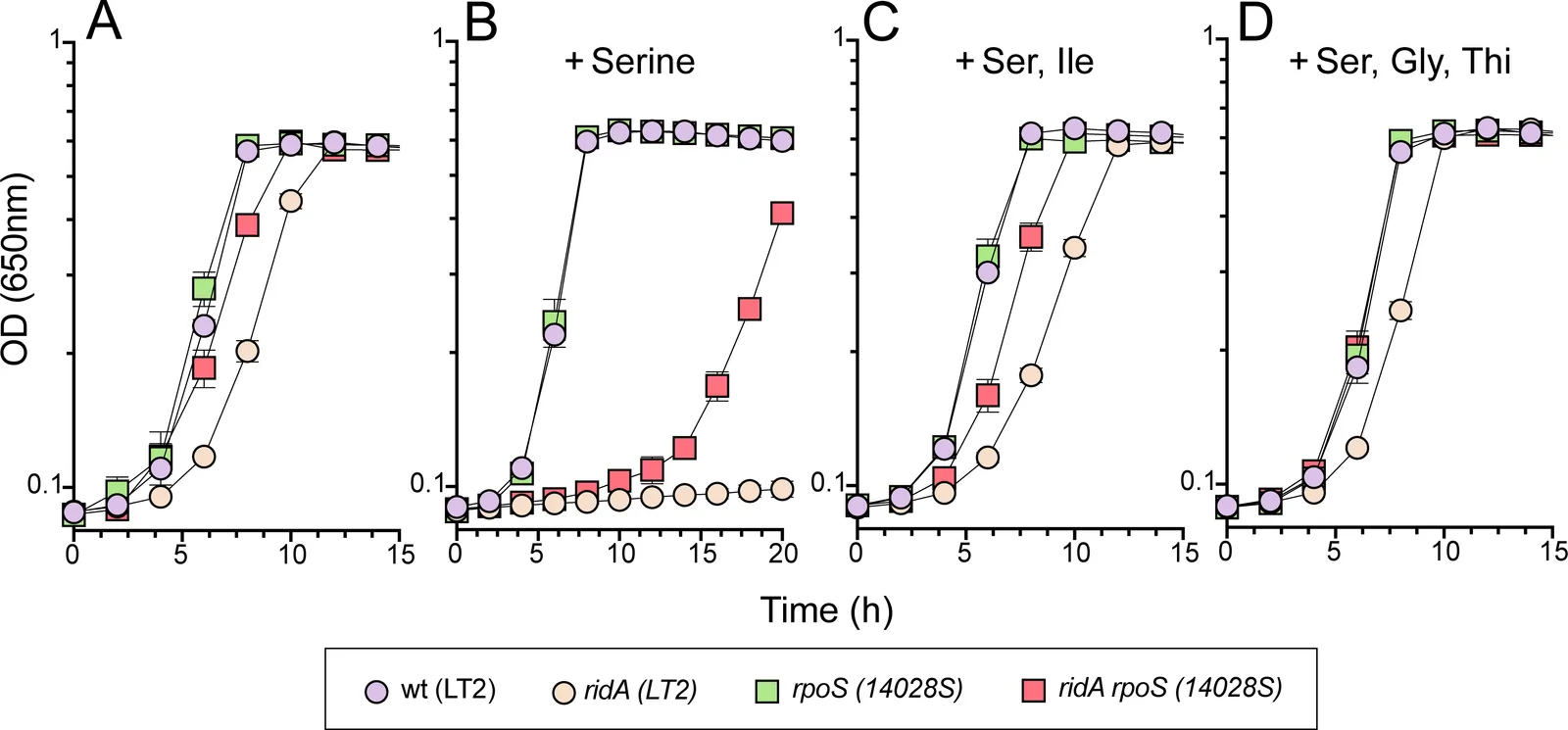
Variation in the *rpoS* allele affects the level of serine sensitivity in a *ridA* mutant. Indicated strains *ridA* (LT2; DM3480), WT (LT2; DM9404), *ridA rpoS* (14028s; DM18737), and its parent strain *rpoS* (14028s; DM18735) were grown at 37°C in glucose minimal medium containing: no addition (**A**); 5 mM L-serine (**B**); 5 mM serine, 1 mM isoleucine (**C**); 5 mM serine, 0.67 mM glycine, 100 nM thiamine (**D**). The data reflect experiments performed in biological triplicate, and error bars represent the standard deviation.

Given the role of RpoS in stress response, it was of interest to probe the role of *rpoS* in the observed growth difference between the two strains. We hypothesized that deleting *rpoS* in the *ridA* (14028s) mutant would phenocopy the *ridA* (LT2) mutant. An *rpoS* insertion mutation was transduced into wild-type (14028s) and *ridA* (14028s) strains. Growth of the resulting strains was compared to WT (LT2) and *ridA* (LT2; Fig. 3). The four strains grew similarly on minimal glucose medium, with the *ridA* (LT2) having a slight growth defect noted previously. When serine (5 mM) was present in the medium, growth was compromised in both strains lacking RidA. A few points were taken from these data. First, the *ridA rpoS* (14028s) double mutant grew in the presence of serine only after 10–15 h and had a lower growth rate than the parental *rpoS* (14028s) mutant. In contrast, the *ridA* (LT2) mutant showed no growth throughout the entire experiment of more than 20 h. Second, the addition of isoleucine to the serine-containing medium restored essentially full growth to both *ridA rpoS* (14028s) and *ridA* (LT2) strains. Finally, the addition of glycine and thiamine to the serine-containing medium restored both *ridA* mutant strains to full growth. The growth patterns observed with the nutritional supplements supported the conclusion that the serine sensitivity in the 14028s background is (i) enhanced by the absence of *rpoS*, (ii) due to 2AA generated by IlvA, (iii) and due primarily to the inhibition of GlyA and IscS by 2AA (44, 45). The *rpoS* insertion mutation used above was moved into wild-type LT2 and a *ridA* (LT2) mutant. Neither of the resulting strains was phenotypically distinguishable from the relevant parental strain (Fig. S3). These data were consistent with the conclusion that the LT2 strain in our laboratory lacks RpoS function.

### Additional growth phenotypes of a *ridA* (14028s) mutant are minimally impacted by RpoS

The results above showed that at least one phenotype caused by the lack of RidA was fully manifested only in the absence of RpoS. If other phenotypes of the *ridA* (LT2) are caused, or affected, by RpoS, it would impact our understanding of the 2AA paradigm and its metabolic components. Thus, we tested additional phenotypes in the 14028s strain derivatives that have been attributed to the accumulation of 2AA.

#### Sensitivity to cysteine and DL-2,3-diaminopropionate

**Fig 4:**
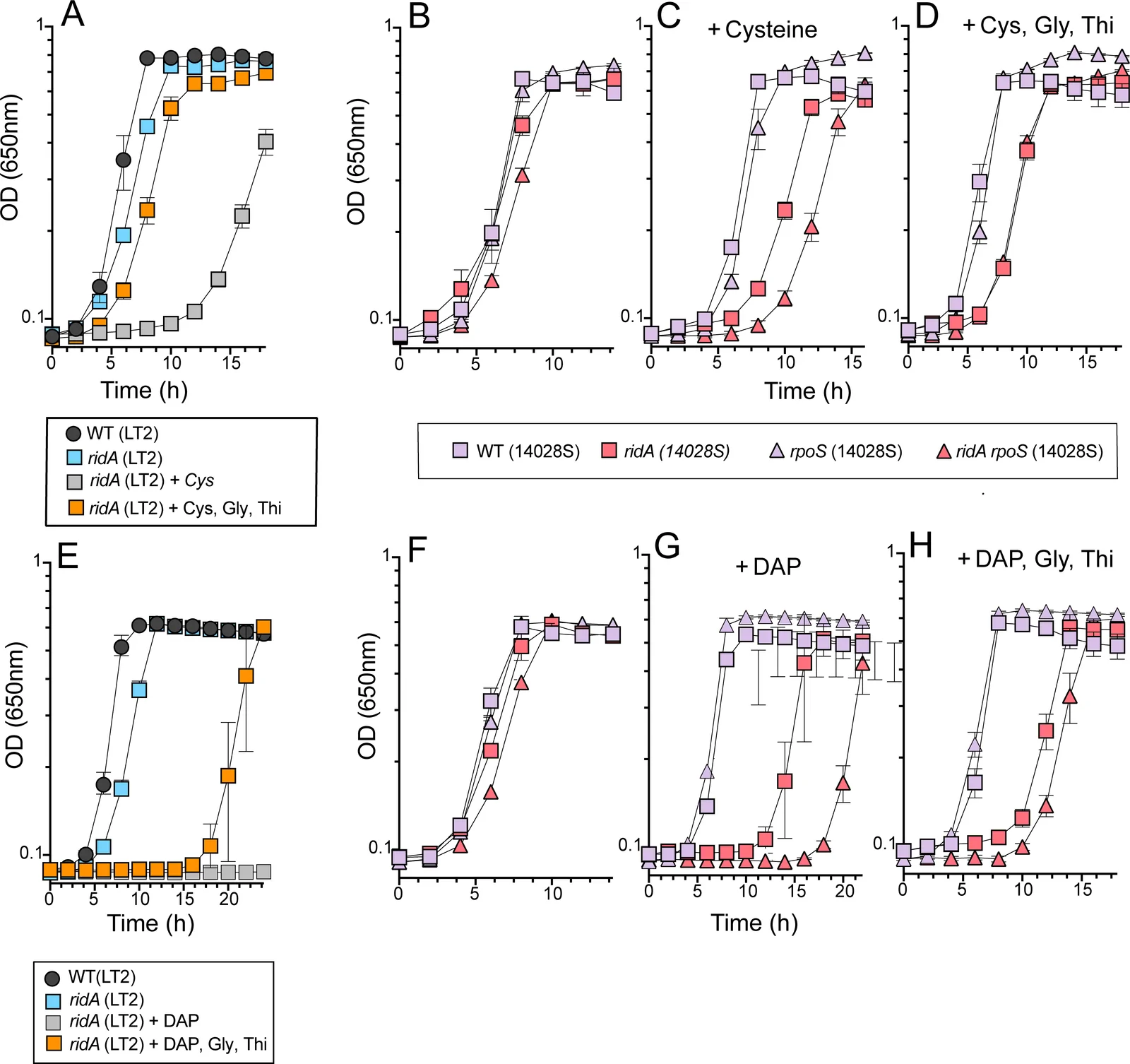
Lesions in *rpoS* and *ridA* affect sensitivity to cysteine and DL-DAP. Strains WT (LT2) and *ridA* (LT2; DM9404 and DM3480, respectively), were grown in minimal medium with indicated supplements (**A and E**). Strains WT (14028s; DM18363), *ridA* (14028s; DM18680), *rpoS* (14028s; DM18735), and *ridA rpoS* (14028s; DM18737) were grown in minimal medium with: (**B**) no supplementation, (**C**) cysteine (0.25 mM), (**D**) cysteine (0.25 mM), glycine (0.67 mM), and thiamine (100 nM); (**F**) no supplementation, (**G**) DL-DAP (0.25 mM), (**H**) DL-DAP (0.25 mM), glycine (0.67 mM), and thiamine (100 nM). Data points represent the mean of three biological replicates; error bars indicate standard deviation.

The PLP-dependent cysteine desulfhydrase (CdsH, EC 2.5.1.47) is used for the detoxification of excess cysteine and has an enzymatic mechanism like that of IlvA (36, 46). CdsH catalyzes the β-elimination of the sulfhydryl group from cysteine, which generates sulfide, ammonia, and 2AA, which is deaminated to pyruvate by RidA. In a *ridA* mutant of LT2, sensitivity to some levels of cysteine is due to 2AA accumulation (47). Similarly, diaminopropionate (DAP) can be toxic for bacteria (48), and the PLP-dependent enzyme DapL (diaminopropionate ammonia-lyase; EC 4.3.1.15) catalyzes the breakdown of DAP, thereby mitigating DAP-induced stress in *S. enterica*. The DapL reaction also proceeds to a 2AA product, and the sensitivity of an LT2 *ridA* mutant to DAP is due to 2AA accumulation (49). The growth phenotypes of *ridA* (14028s) and *ridA rpoS* (14028s) mutants, along with the parental strains, were assessed in the presence of cysteine or DAP. Growth phenotypes of the *ridA* (LT2) mutant strain were confirmed and are shown for comparison (Fig. 4A and E). In glucose minimal medium supplemented with 0.25 mM cysteine, the *ridA* (14028s) mutant had a growth defect, though much less severe than that of the *ridA* (LT2) mutant (Fig. 4A through D). An additional lesion in *rpoS* only slightly decreased growth in the presence of cysteine. Supplementation with glycine and thiamine eliminated the effect of the *rpoS* lesion but did not increase growth of the *ridA* (14028s) mutant. The growth with DL-DAP showed a comparable pattern (Fig. 4E through H). In both experiments, a few points were made from the data. First, a *ridA* mutation in strain 14028s causes a slight sensitivity to both cysteine and DAP. Second, the additional loss of *rpoS* magnifies the growth defect of the *ridA* mutant but not to the extent of the *ridA* (LT2) strain. While the impact of the *rpoS* mutation was eliminated by the addition of glycine and thiamine, these supplements did not impact the effect of the *ridA* mutation. Together, these data suggest that the minimal growth defect of the *ridA* (14028s) mutant was due to target(s) of 2AA other than IscS and GlyA, or an increased sensitivity to cysteine and DL-DAP themselves. In contrast, the loss of *rpoS* exposed 2AA damage to GlyA and IscS, which was circumvented by the addition of thiamine and glycine. Taken together, these data supported the hypothesis that RpoS influences the cellular response to 2AA stress to different degrees and possibly by different mechanism(s) depending on the source of the stressor.

#### Use of pyruvate as carbon source

**Fig 5:**
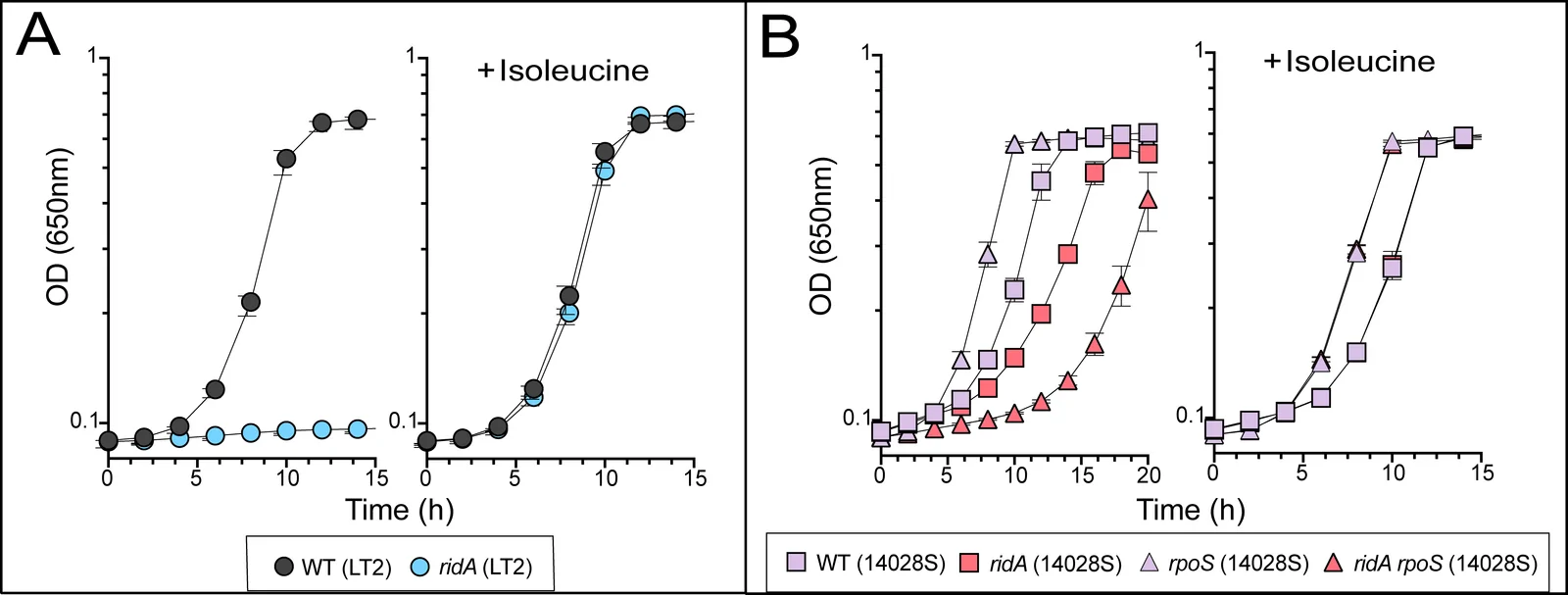
The *rpoS* gene modulates the impact of endogenous serine in a *ridA* mutant. Strains WT (LT2) and *ridA* (LT2; DM9404 and D3480, respectively) (**A**) and *ridA rpoS* (14028s) and *rpoS* (14028s; DM18737 and DM18735, respectively) (**B**) were grown at 37°C in pyruvate (50 mM) minimal medium with no addition or 1 mM isoleucine as indicated. The data reflect experiments performed in biological triplicate, and error bars represent the standard deviation.

A *ridA* (LT2) mutant grows poorly with pyruvate as the sole carbon source due to the combined consequences of endogenously generated 2AA targeting GlyA and IlvE (6, 50). Growth of *ridA* mutants in both the 14028s and LT2 backgrounds, along with their parental strains, was assessed in pyruvate medium (Fig. 5). As expected, the *ridA* (LT2) mutant failed to grow with pyruvate as the sole carbon source (Fig. 5A) (50). In contrast, the *ridA* (14028s) mutant grew only slightly less well than parental strain 14028s. However, in the *rpoS* (14028s) background, a *ridA* mutation compromised growth more significantly, though again not to the level of a *ridA* (LT2) mutant (Fig. 5B). Importantly, the addition of isoleucine reversed the effect of a *ridA* mutation in all cases. Isoleucine feedback inhibits IlvA (51) and, thus, effectively shuts off the major source of 2AA production in *S. enterica*. These data support the conclusion that growth effects caused by the lack of RidA are due to 2AA accumulation and are impacted by a component(s) of the RpoS regulon.

In total, when 2AA generation was independent of exogenous serine, the effect on growth of strain 14028s had qualitatively similar properties: (i) a *ridA* lesion alone was slightly deleterious, (ii) an *rpoS* lesion in combination with the *ridA* mutation further compromised growth, and (iii) restoration of growth with specific nutrients supports the conclusion that a primary cause of the growth defects is 2AA stress. In total, the impact of a *ridA* lesion in 14028s, with or without an *rpoS* mutation, was less severe than in an LT2 strain.

### RpoS-independent consequences of 2AA-dependent stress in strain 14028s

The results above showed that a lesion in *rpoS* could cause or exacerbate multiple growth defects of the *ridA* (14028s) mutant. Two effects of 2AA accumulation that are not measured by growth have been described for a *ridA* (LT2) mutant: motility and BCAT activity (24). In these cases, accumulated 2AA is derived from IlvA acting on endogenous pools of serine and produces a measurable effect not related to growth.

#### Motility

*ridA* (LT2) mutants have compromised swimming motility due to the accumulated 2AA generated from endogenous pools of serine (13). Swimming assays were performed on 0.25% agar plates. On minimal glucose medium, the *ridA* (14028s) had ~60% the motility of the parental 14028s strain (Table 1). This defect was comparable to that reported for a *ridA* (LT2) mutant (13) and repeated here (Table 1). In contrast to the growth phenotypes described above, a *ridA* mutation affected motility to the same extent in both 14028s and LT2 strains. Consistently, the status of *rpoS* had no impact on the motility defect caused by a *ridA* mutation in the 14028s strain (Table 1). The addition of glycine and thiamine did not impact motility of the *ridA* (14028s) strain, while the addition of isoleucine restored full motility (~97% of the parent) to the *ridA* mutant strains. These data support the conclusion that decreased motility is due to the accumulation of 2AA.

**TABLE 1:** 2AA accumulation affects motility independent of RpoS^*a*^

Strain	14028s background Relevant genotype	Supplement	Swim zone (mm)	% motility^*b*^
DM18363	Wild type	None	15.8 ± 1	100
DM18680	*ridA*	None	9.5 ± 1	60
DM18735	*rpoS*	None	14.5 ± 1	100
DM18737	*ridA rpoS*	None	9.3 ± 1	64
DM18363	Wild type	Gly-Thi	19.3 ± 3	100
DM18680	*ridA*	Gly-Thi	11.8 ± 1	61
DM18735	*rpoS*	Gly-Thi	17.2 ± 2	100
DM18737	*ridA rpoS*	Gly-Thi	11.5 ± 1	67
DM18363	Wild type	Ile	13.6 ± 1	100
DM18680	*ridA*	Ile	13.2 ± 1	97
DM18735	*rpoS*	Ile	15.2 ± 1	100
DM18737	*ridA rpoS*	Ile	14.9 ± 1	98

Strain	LT2 background Relevant genotype	Supplement	Swim zone (mm)	% motility
DM9404	Wild type	None	14.6 ± 1	100
DM3480	*ridA*	None	8.1 ± 1	55
DM9404	Wild type	Gly-Thi	19.3 ± 2	100
DM3480	*ridA*	Gly-Thi	12.1 ± 1	63
DM9404	Wild type	Ile	14.9 ± 2	100
DM3480	*ridA*	Ile	14.5 ± 1	97

^*a*^ The diameter of motility swim halos was measured to the nearest millimeter on minimal glucose motility medium (0.25% agar). Supplements were added to the medium as indicated and include: isoleucine (1 mM), glycine (0.67 mM), and thiamine (100 nM). Values reported are the average diameter with standard deviation (SD). Zones were routinely measured for three biological replicates (average of two technical replicates) after incubation for 20 h at 30°C.

^*b*^ % motility represents the strain with a ridA mutation compared to the relevant parental strain.

#### Branched-chain amino acid transaminase activity

Since 2AA damage is post-translational and irreversible, it can be detected as a decrease in activity of the attacked enzymes (8, 16). Transaminase B (IlvE, EC 2.6.1.27) is one target enzyme of 2AA and has been used as a proxy for 2AA accumulation (8, 17). When grown in minimal medium, a *ridA* mutant typically has 60%–70% of the transaminase B activity found in the LT2 parent (8, 17).

IlvE was assayed in appropriate 14028s strains. IlvE activity in the *ridA* (14028s) mutant was decreased when measured in crude extract and compared to the parental WT (14028s) strain (62.4 ± 16 and 107.5 ± 14 nmol/mg/min, respectively). As expected, when cells were grown in the presence of isoleucine, the transaminase activity in *ridA* (14028s) was restored to near wild-type levels (91.9 ± 13 vs 105.4 ± 8 nmol/mg/min, respectively). Unexpectedly, a lesion in *rpoS* increased the IlvE activity in both the WT and *ridA* backgrounds. However, the difference between the *rpoS* and *rpoS ridA* mutants (242.2 ± 19.9 and 173.2 ± 24.1 nmol/mg/min, respectively) parallels that between the strains without the *rpoS* lesion. RpoS does not directly affect the transcription of *ilvE* (52), and the explanation for the increased IlvE activity was not pursued here. In total, data support the conclusion that lack of RpoS can impact the metabolic response(s) of a strain to 2AA stress (e.g., growth phenotype), but it does not significantly impact the level of 2AA stress generated by endogenous serine.

### SdaA is involved in the RpoS-dependent suppression of serine sensitivity

The *ridA* mutant phenotype affected most dramatically by the status of RpoS was sensitivity to exogenous serine. Exogenous serine generates 2AA stress in a *ridA* mutant through the action of the allosterically regulated PLP-dependent serine/threonine deaminase IlvA (8, 53). L-serine can also be deaminated by iron-sulfur cluster-containing serine deaminases, like SdaA (EC 4.3.1.17) (54, 55). In the deaminase reaction catalyzed by SdaA, the 2AA intermediate is not released from the enzyme (56). We considered a scenario in which decreased expression of *sdaA* in an *rpoS* mutant causes more serine to be deaminated by IlvA, resulting in accumulation of higher 2AA levels. In such a scenario, (i) a deletion of *sdaA* would phenocopy an *rpoS* mutation in a *ridA* (14028s) mutant background, and (ii) expression of *sdaA* would be demonstrably lower in the absence of RpoS.

**Fig 6:**
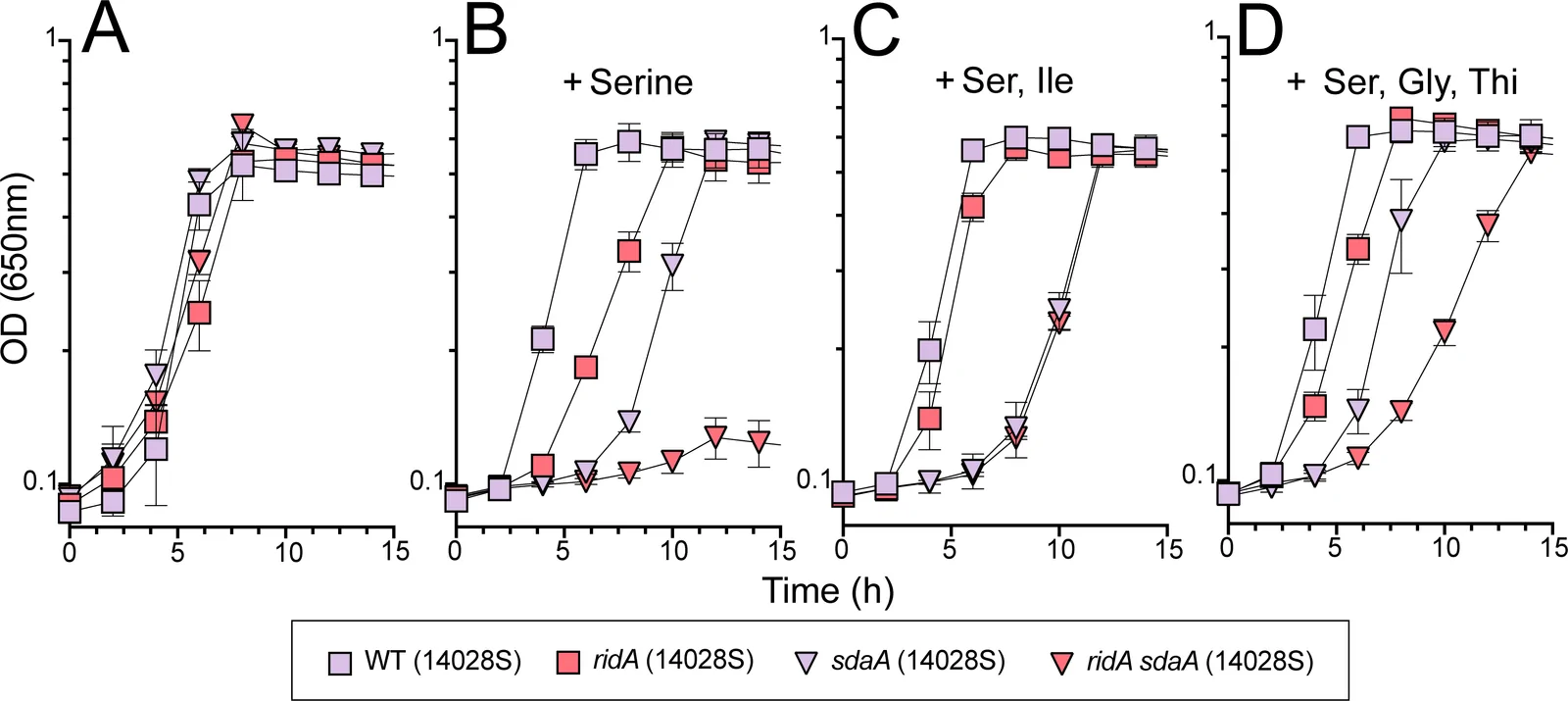
An *sdaA* mutation causes serine sensitivity in a *ridA* (14028s) mutant. Strains WT (14028s; DM18363), *ridA* (14028s; DM18680), *sdaA* (14028s; DM18773), and *ridA sdaA* (14028s; DM18774) were grown at 37°C in glucose minimal medium with: no addition (**A**), 5 mM L-serine (**B**), 5 mM serine, and 1 mM isoleucine (**C**), 5 mM serine, 0.67 mM glycine, and 100 nM thiamine (**D**). The data reflect experiments performed in biological triplicate, and error bars represent the standard deviation.

An *sdaA* insertion mutation was transduced into WT (14028s) and *ridA* (14028s), and the resulting strains were evaluated for serine sensitivity. The *sdaA ridA* (14028s) mutant failed to grow in the presence of exogenous serine, consistent with the scenario suggested above (Fig. 6). The impact of a *ridA* mutation on strains with or without an *sdaA* lesion was eliminated by the addition of isoleucine. These results again supported the conclusion that IlvA-generated 2AA was responsible for the serine-dependent growth defect caused by a *ridA* mutation. Furthermore, the data supported a scenario in which 2AA accumulation is increased when SdaA is absent. This hypothesis is further supported by the positive impact of glycine and thiamine on the growth of the *sdaA ridA* (14028s) mutant (Fig. 6D). The latter result is consistent with a growth defect caused by lower IscS and/or GlyA activity. The slight serine sensitivity of the *sdaA* mutants in the presence of isoleucine indicates a sensitivity to serine independent of 2AA, which has been noted in the literature (57, 58). The data show that an *sdaA ridA* (14028s) strain is more sensitive to exogenous serine than a *ridA rpoS* (14028s) strain (Fig. 3B vs 6B), suggesting that the basal level of SdaA, present in the absence of *rpoS*, contributes to preventing 2AA stress from exogenous serine.

### Expression of *sdaA* is decreased in an *rpoS* mutant

Several genome-wide transcriptome studies have suggested that RpoS activates expression of *sdaA* (28, 52, 59). Validation of the results from global experiments was sought with real-time qRT-PCR. Expression of *sdaA* was determined in three relevant strains that differed in *rpoS* (Table 2). Expression of *sdaA* was ~15-fold lower in the *rpoS* (14028s) strain than in WT (14028s). Consistently, the level of *sdaA* expression in WT (LT2) was more similar to that found in the *rpoS* (14028s) strain than in WT (14028s). These data validated results from global analysis and support the conclusion that in a strain lacking RpoS, there is less SdaA.

**TABLE 2:** Expression of *sdaA* is decreased in the absence of RpoS^*a*^

Strain		Relative expression (2^−ΔΔCt^ ± standard deviation)	*P*-value (Welch’s test)
DM9404	WT (LT2)	0.61 ± 0.06	0.0004
DM18363	WT (14028s)	4.95 ± 0.36	
DM18735	*rpoS* (14028s)	0.34 ± 0.03	

^*a*^ Expression of *sdaA *was measured using real-time PCR. Samples were grown in biological triplicate in glucose minimal media supplemented with 5 mM serine at 37°C. Expression of *sdaA *from each strain was normalized to *gyrA* and then compared to one another using a two-tailed Welch’s T-test.

### Static growth uncovers another RpoS-dependent effect on 2AA stress

**Fig 7:**
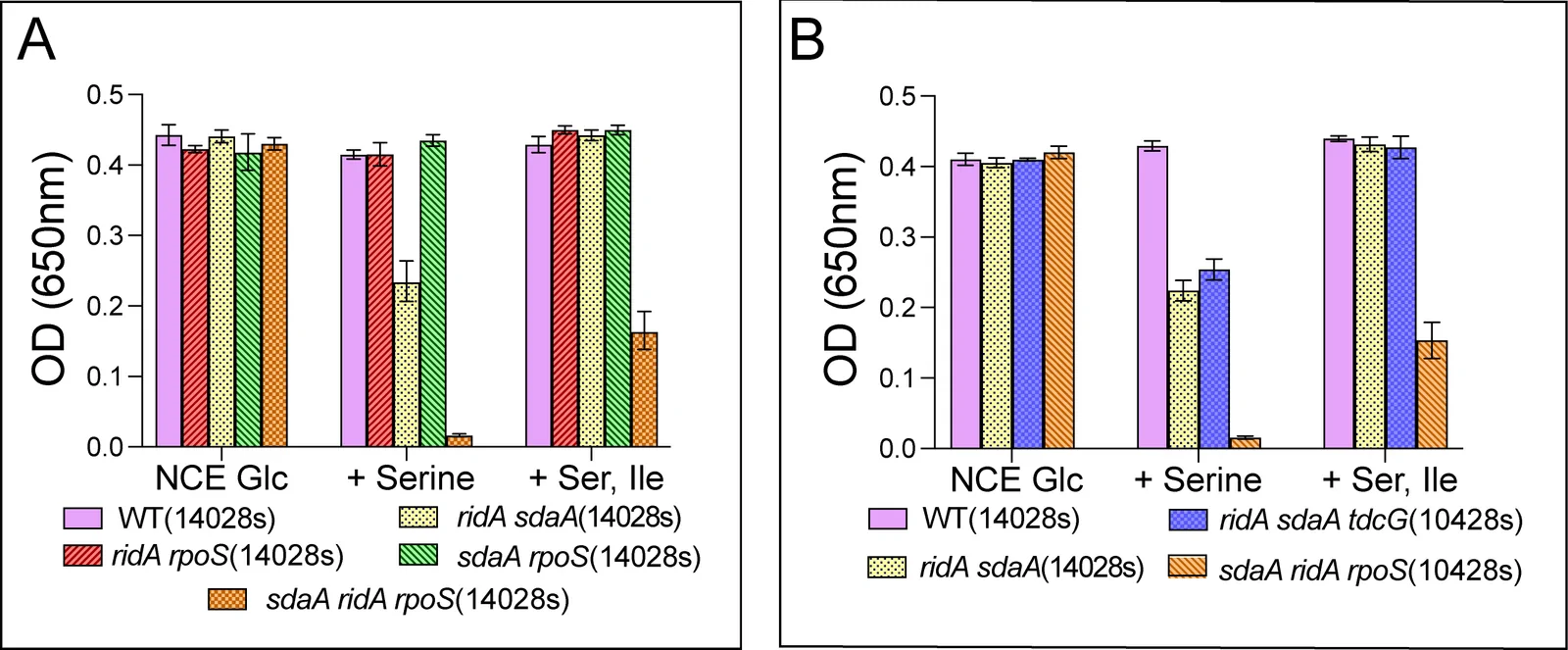
SdaA, but not TdcG, contributes to serine resistance in static conditions. 14028s strains (**A**) WT (DM18363), *ridA rpoS* (DM1873), *ridA sdaA* (DM18774), *sdaA rpoS* (DM18775), and *sdaA ridA rpoS* (DM18787), and (**B**) WT (DM18363), *ridA sdaA* (DM18774), *ridA sdaA tdcG* (DM19085), and *sdaA ridA rpoS* (DM18787) were grown statically for 24 h in No-carbon E (NCE) glucose medium with no additions, with serine (5 mM), or with both serine (5 mM) and isoleucine (1 mM) as indicated. Data points represent the mean of three biological replicates, with error bars indicating standard deviation.

SdaA is an iron-sulfur cluster protein and, as such, is sensitive to oxidative damage that occurs during growth under aerated conditions (55, 59, 60). Thus, the level of *sdaA* transcribed in the absence of RpoS is expected to generate more dehydratase activity when cells are grown in sufficiently low oxygen. This possibility suggests that the *rpoS ridA* (14028s) mutant might be more resistant to serine under static growth conditions. This general thinking was supported by the finding that a *ridA* (LT2) strain is not sensitive to serine when grown in static conditions (Christopherson and Downs, unpublished). Static growth conditions are demonstrably low in oxygen based on the ability of a *metE* mutant strain to grow without methionine (61). In fact, the *ridA rpoS* (14028s) mutant strain reached full density in the presence of serine (5 mM) after 12 h of growth in static conditions (Fig. 7). In the simplest scenario, the *sdaA ridA* (14028s) mutant strain would be sensitive to serine in both shaking and static conditions. Under static conditions, the *sdaA ridA* (14028s) reached a density of OD_650_ 0.23 ± 0.02 in minimal medium with serine after 12 h. This yield was ~55% of that reached in minimal medium (OD_650_ 0.44 ± 0.01; Fig. 7A). The addition of isoleucine restored 100% yield to the *sdaA ridA* (14028s) strain in the presence of serine (OD_650_ 0.44 ± 0.01), implicating IlvA-derived 2AA in the growth defect. However, in the same serine-containing medium, the triple *sdaA ridA rpoS* (14028s) mutant reached less than 5% of the density reached in minimal medium (OD_650_ 0.02 and 0.43 ± 0.01, respectively). In this strain, the addition of isoleucine increased yield to 40% of that achieved in minimal medium (OD_650_ 0.16 ± 0.02). These results showed that there was an RpoS-dependent, SdaA-independent contribution to growth in low oxygen when serine is present. The *tdcG* gene encodes an Fe-S-dependent serine/threonine dehydratase and is expected to be expressed in low-oxygen conditions (62). If TdcG contributes to serine deamination under static conditions, a *tdcG* mutation is expected to mimic the effect of an *rpoS* mutation in the *sdaA ridA* (14028s) strain. However, data in Fig. 7B showed that the *sdaA ridA* (14028s) and *tdcG sdaA ridA* (14028s) mutants had similar final densities in the presence of serine (OD_650_ 0.22 ± 0.01 and 0.25 ± 0.01, respectively). From these data, we concluded that in aerated conditions, SdaA activity contributes to the serine resistance of a *ridA* (14028s) strain, but in oxygen-limiting conditions, there is an additional RpoS-dependent contributor(s). The partial restoration of growth by isoleucine in the presence of serine suggests that there are factors beyond the IlvA-dependent 2AA stress that compromise growth in the *sdaA ridA rpoS* (14028s) strain.

### SdaA is specifically involved with exogenous serine-generated sensitivity

**Fig 8:**
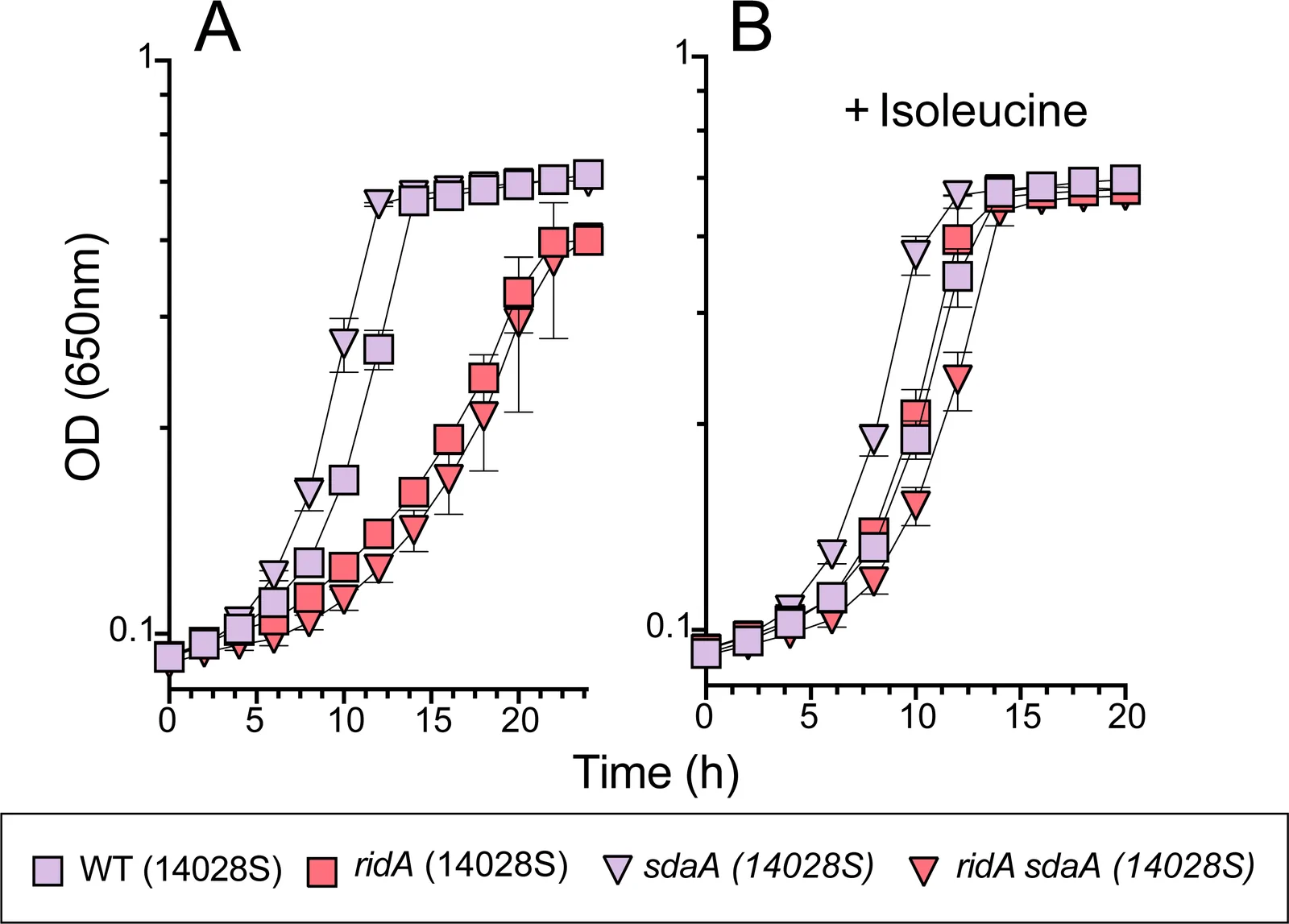
An *sdaA* mutation does not affect 2AA-dependent inhibition of pyruvate growth. The *ridA* (14028s; DM18680) and *ridA sdaA* (14028s; DM18774) mutants, along with their parental strains, WT (14028s; DM18363) and *sdaA* (14028s; DM18773), respectively, were grown at 37°C in NCE 50 mM pyruvate minimal media with no supplements (**A**) and 1 mM isoleucine (**B**). Data points represent the mean of biological triplicates, and error bars indicate standard deviation.

The data above showed that the status of SdaA impacted 2AA-dependent phenotypes that relied on exogenous serine, specifically under aerated growth conditions. It was formally possible that SdaA activity could decrease the amount of free 2AA generated from endogenous serine. If SdaA competes with IlvA at low serine concentrations, an *sdaA ridA* (14028s) mutant might be expected to have increased 2AA accumulation that would manifest as a growth defect on pyruvate, decreased motility, and/or decreased IlvE activity compared to the *ridA* single mutant. A lesion in *sdaA* had no effect on the growth of the *ridA* (14028s) mutant in No-carbon E (NCE) pyruvate media (Fig. 8). Similarly, an *sdaA* mutation did not decrease the IlvE activity of a *ridA* (14028s) mutant (data not shown). These data suggest that SdaA does not compete with IlvA for serine at concentrations found endogenously.

## DISCUSSION

It has been well documented that when the metabolic network of a bacterium is perturbed, whether by external stress, mutation, or genomic differences, the impact can be minimized by remodeling the distribution of metabolite flux. It follows that apparently small differences in genome content can generate network changes that result in variant growth behaviors. Our still incomplete understanding of metabolic network architecture means that the results of perturbations are not easily predicted. This is true not only for closely related species (e.g., *S. enterica* and *E. coli*) but also for strains of the same species, as shown herein. *S. enterica* strains LT2 and 14028s carry distinct alleles of *rpoS*, and differences in virulence between the two strains are attributed to the different status of RpoS.

Stark differences in phenotypes caused by a *ridA* mutation in the 14028s background compared to those characterized in the LT2 strain led us to question a role for RpoS in the response to 2AA stress. To address the simple scenario that RpoS was responsible for the phenotypic differences between strains, a *ridA rpoS* double mutant of 14028s was constructed. While the *ridA rpoS* (14028s) mutant did not directly phenocopy a *ridA* (LT2) mutant, it differed significantly from the single *ridA* (14028s) mutant. This result supported the conclusion that the status of RpoS impacts the cellular response to 2AA stress and that the cause of the distinct manifestations of 2AA stress in LT2 and 14028s strain backgrounds is multi-factorial, with RpoS being one contributor.

Two effects of a *ridA* mutation appeared to be unaffected by the status of *rpoS* in 14028s. A *ridA* mutation decreased the activity of IlvE, with or without a lesion in *rpoS*. This result supported the conclusion that 2AA accumulation was not dramatically altered by the lack of RpoS. Additionally, the motility defect characteristic of a *ridA* mutant was not affected by the status of *rpoS*. Unlike growth defects attributed to a *ridA* mutation, the mechanism by which 2AA causes a motility defect is unclear (13). While *ridA* mutants have decreased expression of flagellar genes, the defined targets of 2AA are PLP-dependent enzymes, and there are none obviously associated with motility. The finding that RpoS has no effect on the motility of a *ridA* mutant is consistent with the 2AA acting directly on a process required for motility or its regulation. This scenario would group the IlvE activity and the motility as distinct from the growth defects, which can be impacted by the subtle remodeling of the metabolic network expected by the breadth of the RpoS regulon.

An *rpoS* lesion dramatically increased the sensitivity of a *ridA* (14028s) strain to serine addition. This observation suggested that a member of the RpoS regulon was responsible for preventing exogenous serine from affecting growth of the *ridA* (14028s) mutant. Alleviation of serine sensitivity by isoleucine confirmed that the activity of IlvA on serine was responsible for the problematic 2AA accumulation. We sought an enzyme that acted on serine, did not generate free 2AA, and was activated by RpoS as a possible explanation for the growth difference between *ridA* (14028s) and *ridA rpoS* (14028s) with serine. SdaA satisfied these criteria. As an Fe-S cluster serine/threonine dehydratase, SdaA does not release the 2AA from the enzyme (56). Global data sets and the targeted RT-PCR herein confirmed that this *sdaA* gene is part of the RpoS regulon. The extreme serine sensitivity of the *ridA sdaA* (14028s) mutant supports our hypothesis and suggests that the basal level of *sdaA* transcribed in the absence of *rpoS* is responsible for weak (as opposed to no) growth with serine.

Characteristics of SdaA compared to IlvA predicted that aeration would impact the effect of exogenous serine on a *ridA* mutant. In fact, the *ridA rpoS* (14028s) was not sensitive to serine if growth was under static conditions. This result was consistent with a previous observation of *ridA* (LT2) growth (Christopherson and Downs, unpublished) and suggested that, as an Fe-S cluster protein, SdaA was more active in the absence of damaging ROS generated during aeration. The simple scenario suggested a *ridA sdaA* (14028s) mutant would be sensitive to serine under static conditions. The *ridA sdaA* (14028s) mutant was only slightly sensitive to serine under static growth conditions. Introduction of an *rpoS* lesion further reduced growth in the presence of serine, indicating that a component of the RpoS regulon, in addition to *sdaA*, was involved in detoxifying serine under limiting oxygen conditions. Deletion of the possible candidate *tdcG* did not phenocopy the *rpoS* lesion, leaving the role of RpoS in this situation unclear. Thus, resistance of a *ridA* mutant to exogenous serine has multiple components, one due to SdaA (aerobically), and a second that is *rpoS*-dependent, but functional only under no/low oxygen conditions.

In total, the data herein expanded our understanding of the 2AA stress paradigm and exploited current knowledge to explore the growth consequences of altering the metabolic network structure. The results emphasize both the overall similarity and the subtle differences in flux and/or regulation between the LT2 and 14028s *S. enterica* strains. The *rpoS* locus is the cause of virulence differences between these strains, and the data herein supported a role for RpoS in the different responses to 2AA stress. However, the impact of eliminating RpoS was not sufficient to be the sole explanation of differences between LT2 and 14028s in various metabolic properties. The framework of 2AA stress in 14028s aligns with that in LT2, but the severity of resulting growth defects differs. These differences reflect subtle variation in the architecture of the metabolic network caused by differences in the genomes that might otherwise be dismissed as insignificant. Slight differences in enzyme kinetics, transcriptional regulation, etc., can result in the fitness differences observed in various conditions. The results here identified a role for SdaA in detoxifying serine in a *ridA* mutant. This finding is an example of two catalytically similar enzymes that can have differing roles in fitness depending on the conditions. The two serine dehydratases involved have distinct limitations; the PLP-dependent enzyme (IlvA) produces a reactive metabolite, making it detrimental in the absence of RidA. In contrast, the Fe-S cluster enzyme (SdaA) is sensitive to oxidative damage, making it a liability in the aerated environment.

Additional studies are required to identify the RpoS-dependent component(s) that detoxify serine under low oxygen. In addition, significant work will be required to rigorously map the network alterations between strains LT2 and 14028s and the mechanism(s) generating them. Importantly, phenotypic outputs represent the sum of many small, subtle changes that can result from pathway integration and flux alterations that often cannot be clearly articulated or predicted, even from genome sequences. The 2AA stress paradigm and the associated cellular responses provide a useful model system to further our understanding of mechanisms available to alter metabolic network structure.

## MATERIALS AND METHODS

### Strains, media, and chemicals

The strains used in this study are derivatives of *Salmonella enterica* serovar Typhimurium LT2 or *Salmonella enterica* serovar Typhimurium 14028s and are listed in Table S1. *S. enterica* strains were routinely grown at 37°C in Nutrient Broth (NB) containing 8 g/L of base medium (Difco) and 5 g/L NaCl. Minimal medium was NCE medium supplemented with 11 mM glucose or 50 mM pyruvate, 0.1 mM MgSO_4_, and trace elements (63). Additional supplements were included as indicated in the text and included L-isoleucine (1 mM), L-glycine (0.67 mM), thiamine (100 nM), L-cysteine (0.25 mM), DL-DAP (0.25 mM), L-arabinose (0.2% [wt/vol]), and L-serine (5 mM). When required, antibiotics were added: kanamycin (Km; 50 μg/mL), ampicillin (Amp; 150 μg/mL), tetracycline (Tc; 20 μg/mL), and chloramphenicol (Cm; 20 μg/mL). Chemicals were obtained from MilliporeSigma (St. Louis, MO).

### Genetic techniques

Strains were constructed by transduction using the high-frequency general transducing mutant of bacteriophage P22 (HT105/1 *int-201*) (64). Methods for transduction, purification, and identification of phage-free transductants were as described elsewhere (65).

### Whole-genome sequencing and bioinformatic analysis

Strains to be sequenced were grown in 2 mL of NB overnight at 37°C, pelleted, and stored at −80°C. Illumina sequencing was performed by SeqCenter company. The resulting FASTQ files were cleaned, trimmed, and aligned using Breseq software (43). The amino acid sequences resulting from the WGS data were analyzed with the *Transition and Translator* online tool using the detect stop codon feature (66).

### Growth analyses

Overnight cultures were initiated using single colonies and grown in 2 mL NB medium with shaking (210 RPM) at 37°C. Cells were harvested by centrifugation and resuspended in an equal volume of sterile saline. *Aerated growth*—5 μL of the cell suspension was inoculated into 195 μL of the specified medium. Growth was monitored in 96-well microtiter plates by measuring the optical density (OD_650 nm_) every 30 min using a BioTek ELx808 plate reader (BioTek Instruments, Winooski, VT) set to a slow shaking speed (210 RPM). Data were analyzed and plotted using GraphPad Prism version 8.0.1. All growth experiments were conducted in three independent biological replicates. *Static growth*—100 µL of the saline cell suspension was inoculated into 5 mL of either glucose minimal medium or glucose minimal medium supplemented with serine (5 mM) in 1.25 × 9.5 cm borosilicate tubes. The tubes were incubated at 37°C without shaking for 24 h. Cultures were vortexed to mix prior to reading the optical density (OD_650 nm_) using the GENESYS 30 Visible Spectrophotometer (Thermo Scientific). Growth of a *metE* mutant without supplementation of methionine confirmed that this condition lacked oxygen (data not shown) (61).

### Transaminase B (IlvE) assay

Cultures were grown overnight in 2 mL of NB at 37°C with shaking (210 RPM). Cells were collected by centrifugation and resuspended in an equal volume of sterile saline. An aliquot of the saline suspension (100 μL) was inoculated into 10 mL of glucose minimal medium and the same medium supplemented with isoleucine (1 mM). When cultures reached an optical density (OD_650 nm_) of ~1, cells were pelleted, resuspended in 10 mL of NCE medium, pelleted a second time, and stored at –80°C until use. For assays, frozen cell pellets were resuspended in 450 μL potassium phosphate buffer (50 mM, pH 7.5) and disrupted by mechanical lysis with a Model CL-18 Sonicator (QSonica, Newtown, CT) pulsed 2 s on, 2 s off for 30 s at 40% amplitude. Lysates were clarified by centrifugation at 17,000 × *g* for 20 min at 4°C. The protein concentration of each lysate was determined using the Pierce bicinchoninic acid (BCA) assay kit. Transaminase B activity was assayed as described (8, 37). Briefly, cell-free extract (100 μL) was added to a reaction mixture containing α-ketoglutarate (10 mM) in potassium phosphate buffer (50 mM, pH 7.5). The reaction was initiated with the addition of L-isoleucine (to 20 mM) and incubated at 37°C for 20 min. Product 2-ketomethylvalerate (2KMV) was derivatized with 2,4-dinitrophenylhydrazine (200 μL) to yield a hydrazone, which was treated with 0.5 N HCl (0.5 mL). The resulting organic layer was removed and combined with 1.5 N NaOH (1 mL) to allow formation of an aqueous layer containing a chromophore with an absorbance at 540 nm. The aqueous layer was extracted, and absorbance (540 nm) was measured using a SpectraMax M2 microplate reader (Molecular Devices, San Jose, CA). Activity is reported as the ratio between 2KMV absorbance at 540 nm (A540) and sample biomass measured at 562 nm (A562). Data plotting and statistical analyses (mean and standard error of three biological replicates) were conducted using GraphPad Prism version 11.0.1.

### Motility assay

Motility was assessed on minimal medium motility plates (0.25% agar) with 11 mM D-glucose as the sole carbon source. Nutrients were added to the medium prior to pouring plates at the indicated concentrations. Plates were prepared, allowed to solidify at room temperature, and used approximately 5 h later. Relevant strains were grown to full density in NB, and a 2 μL aliquot was dispensed onto the center of a motility plate. Plates were incubated at 30°C inside a sealed 6-quart plastic container to maintain consistent moisture levels. Motility halos were measured to the nearest millimeter after 20 h. Technical replicates (using individual plates) were prepared for each of three biological replicates. Final data are presented as mean halo diameter (mm) ± standard deviation.

### RNA extraction and cDNA conversion

Single colonies of DM9404 (WT [LT2]), DM18363 (WT [14028s]), and DM18735 (*rpoS* [14028s]) were used to inoculate 2 mL NB in biological triplicate. Cultures were incubated at 37°C with shaking (210 RPM) overnight. Cells were pelleted and resuspended in 2 mL saline (0.85%) before sub-culturing (1:100) into 10 mL NCE medium supplemented with glucose (11 mM) and serine (5 mM). Cultures were incubated at 37°C with shaking (210 RPM) until they reached an average OD_650 nm_ ~ 1.0. Cells were harvested via centrifugation at 17,000 × *g* for 5 min (4°C), and the pellets were flash frozen in liquid nitrogen and stored at −80°C until use.

RNA was extracted using the RNASnap method described previously (67). Cell pellets were resuspended in 150 μL boil solution {EDTA (0.5 M, pH 8), SDS (0.025%, [wt/vol]), β-mercaptoethanol (1%), formamide (95%, [vol/vol])} and lysed by incubation at 95°C for 7 min. Lysates were isolated by centrifugation at 17,000 × *g* for 5 min, and 100 μL of the supernatant was transferred to a new tube containing 400 μL of H_2_O and 50 μL sodium acetate solution (3 M). Nucleic acids were precipitated by adding 1,600 μL of 100% cold ethanol before incubating at −80°C for 1 h. Nucleic acids were then pelleted by centrifugation at 16,000 × *g* for 30 min at 4°C, washed with 300 μL of 70% cold ethanol, and pelleted again at 8,000 × *g* for 8 min at 4°C. Supernatants were removed, pellets were air-dried, and resuspended in 100 μL of H_2_O. Water-insoluble components were removed by pelleting at 16,000 × *g* for 1 min, and 90 µL of the supernatant was treated with DNase using the TURBO DNase DNA-free kit per manufacturer’s instructions (Invitrogen, Waltham, MA). RNA was purified by ethanol precipitation as described above.

RNA was quantified using the Qubit RNA Broad Range kit (Invitrogen, Waltham, MA). All RNA samples possessed an RNA integrity and quality (IQ) score of at least 8.5 as determined by the Qubit RNA IQ assay kit (Invitrogen, Waltham, MA). RNA samples (0.5 μg) were immediately reverse transcribed into cDNA using the iScript cDNA synthesis kit per manufacturer’s instructions (BioRad, Hercules, CA), and cDNA was stored at −20°C until use.

### Real-time PCR assay

RT-qPCR was performed using a 96-well optical plate and a 7500 Fast Real-Time PCR system (Applied Biosystems, Waltham, MA). Each 20 μL RT-qPCR reaction contained 1× Fast SYBR Green master mix (Applied Biosystems, Waltham, MA), 0.5 μM of each gene-specific primer (Table S2), and 20 ng of cDNA. A melting curve analysis was included to ensure specificity of the reaction. For each gene, the average cycle threshold (Ct) of each biological replicate was calculated by taking the mean of three technical replicates. Expression of *sdaA* was normalized to the reference gene, *gyrA*, by calculating ΔCt for each of the three biological replicates. To determine the fold change in *sdaA* expression between strains, the comparative threshold cycle (ΔΔCt) method was utilized (68). The resulting 2^−ΔΔCt^ values for each strain were compared using a two-tailed Welch’s *t*-test in Prism 11.0.1 (GraphPad).

## Data Availability

All relevant data are included in the content of this manuscript.

## SUPPLEMENTAL MATERIAL

Supplemental material jb.00390-26-s0001.pdf Tables S1 and S2; Fig. S1 to S3.

ASM does not own the copyrights to Supplemental Material that may be linked to, or accessed through, an article. The authors have granted ASM a non-exclusive, world-wide license to publish the Supplemental Material files. Please contact the corresponding author directly for reuse.
