# Supplementary material for: The status of RpoS impacts the 2-aminoacrylate stress paradigm in ridA mutants of Salmonella enterica serovar Typhimurium
Source: J Bacteriol. 2026 Sep 18;208(10):e00390-26. doi: 10.1128/jb.00390-26 (PMC13689558; doi:10.1128/jb.00390-26)
Supplement: Supplementary file 1 [file jb.00390-26-s0001.pdf]

SUPPLEMENTAL MATERIAL

The status of RpoS impacts the 2-aminoacrylate stress paradigm in *ridA* mutants of *Salmonella enterica* serovar Typhimurium

Andrea Giuliano[1], Jessica K. Novak, and Diana M. Downs*

Department of Microbiology
University of Georgia, Athens, GA, USA.

Running title: RpoS impacts growth of *ridA* mutants

Key Words: RpoS, RidA, 2-aminoacrylate, metabolic stress

***Correspondence:**

Dr. Diana M. Downs
Department of Microbiology, University of Georgia
Athens, GA, USA, 30602
Email: dmdowns@uga.edu

[1]**Current address:**
Department of Biological and Environmental Sciences and Technologies (DiSTeBA), University of Salento, Lecce, 73100, Italy
Department of Medical Biotechnology, University of Siena, Siena, 53100, Italy

Table S1. Strains utilized in this study.

Strain	Genotype	Source
**14028s strain background**		
DM18363	Wild-type	Laboratory collection
DM18680	*ridA3*::*Mu*dJ[a]	This study
DM18735	*rpoS1117*::Amp	This study
DM18737	*ridA3*::*Mu*dJ *rpoS1117*::Amp	This study
DM18773	*sdaA1*::Tn*10*(d)Tc[b]	This study
DM18774	*sdaA1*::Tn*10*(d)Tc *ridA3*::*Mu*dJ	This study
DM18775	*sdaA1*::Tn*10*(d)Tc *rpoS1117*::Amp	This study
DM18787	*sdaA1*::Tn*10*(d)Tc *ridA3*::*Mu*dJ *rpoS1117*::Amp	This study
DM19068	*ridA4*::Cm	This study
DM19075	*ridA*::Cm *sdaA1*::Tn*10*(d)Tc	This study
DM19076	*tdcG*::Km(*nt3423281-3424584*)[c]	This study
DM19084	*ridA4*::Cm *tdcG*::Km(*nt3423281-3424584*)	This study
DM19085	*ridA4*::Cm *sdaA1*::Tn*10*(d)Tc *tdcG*::Km(*nt3423281-3424584*)	This study
**LT2 genetic background**		
DM9404	Wild-type	Laboratory collection
DM3480	*ridA3*::*Mu*dJ	Laboratory collection
DM15340	*rpoS1117*::Amp	Laboratory collection
DM15341	*rpoS1117*::Amp *ridA3*::*Mu*dJ	Laboratory collection

[a] *Mu*dJ refers to the *Mu*dJ1734 insertion element (Castilho et al., 1984).
[b] Tn*10*d refers to the transposition-defective mini-Tn*10* (Tn*10*Δ16Δ17) and encodes resistance to tetracycline (Tc) (Way et al., 1984).
[c.] Strain from the single-gene deletion collection in *Salmonella enterica* sv Typhimurium 14028s (Porwollik et al., 2024) was used as donor in transduction to construct stains with this insertion.

Table S2. Primers utilized in this study.

Primer name	Sequence (5' to 3')
gyrA_qPCR_F	GGGCGTTATCCTTATCCGCAC
gyrA.qPCR_R	GCCGTCGATAGCGTCGAGTTC
sdaA-F(qPCR)	GCGGTACTGGCTTACTACGAC
sdaA-R(qPCR)	CGTTCATCTTATACAGCGCGC

Figure S1. A *ridA* (14028s) strain is not sensitive to serine. Final OD_{650nm} of relevant strains was determined after 20 hours of growth. Medium was glucose minimal medium supplemented the indicated concentration of serine, with (B) or without (A) the addition of 1 mM isoleucine. Data points represent the mean of three biological replicates with error bars indicating standard deviation, except for the condition noted with an asterisk. In this latter case, two biological replicas were used.

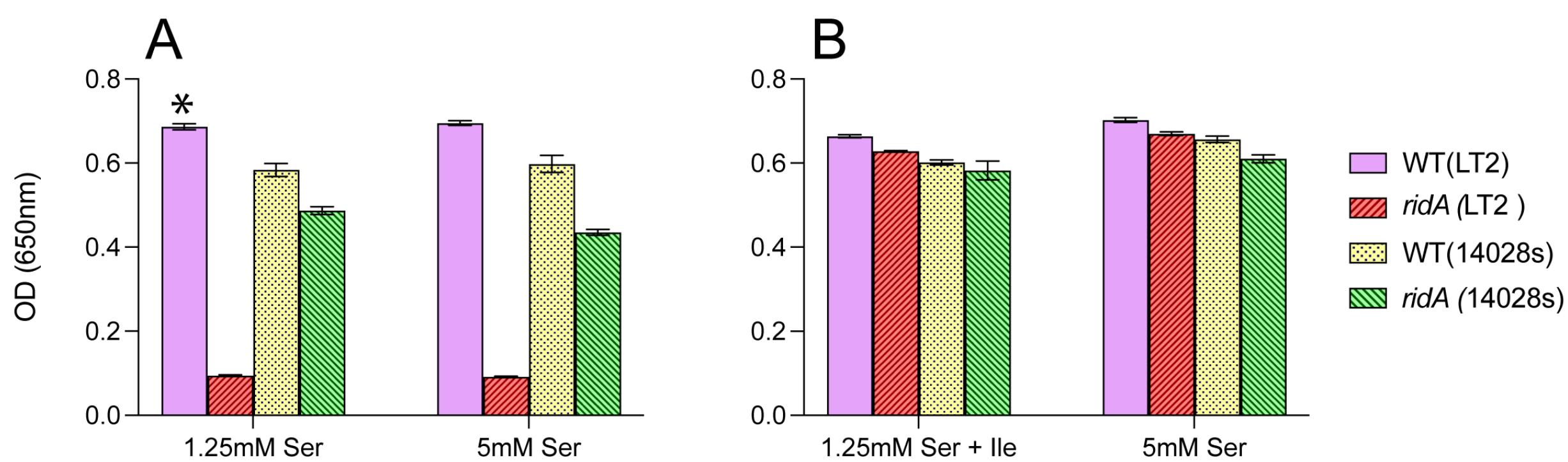

Figure S2. The *rpoS* gene from LT2 contains an 8-bp deletion which likely impairs the DNA-binding domain of the encoded protein. (A) BLAST alignment between the wild-type rpoS nucleotide sequence and the 8-bp deletion variant. (B) UniProt feature viewer for RpoS (LT2, P0DM81) highlighting the C-terminal DNA-binding domain.

A

```
Score            Expect  Identities      Gaps       Strand
1768 bits(957)   0.0     977/985(99%)    8/985(0%)  Plus/Plus

Query  1    TTGAGTCAGAATACGCTGAAAGTTCATGATTTAAATGAAGACGCGGAATTTGATGAGAAC  60
Sbjct  1    ............................................................  60

Query  61   GGAGTAGAGGCTTTTGACGAAAAAGCCTTGAGTGAAGAGGAACCCAGTGATAACGACCTG  120
Sbjct  61   ............................................................  120

Query  121  GCTGAAGAAGAGCTGTTATCGCAAGGGGCCACACAGCGTGTGTTGGACGCGACTCAGCTT  180
Sbjct  121  ............................................................  180

Query  181  TACCTTGGTGAGATTGGGTATTCACCACTGTTAACAGCCGAAGAAGAAGTCTATTTTGCG  240
Sbjct  181  ............................................................  240

Query  241  CGTCGCGCACTGCGTGGAGATGTCGCTTCTCGCCGTCGCATGATTGAGAGTAACCTGCGT  300
Sbjct  241  ............................................................  300

Query  301  CTGGTGGTAAAAATTGCCCGCCGTTATGGCAATCGTGGACTGGCGTTGCTGTGAAGAGGG  360
Sbjct  301  ............................................................  360

Query  361  CAACCTGGGGCTTATCCGTGCAGTCGAGAAGTTTGACCCGGAACGCGGGTTCCGCTTCTC  420
Sbjct  361  ............................................................  420

Query  421  AACATACGCAACCTGGTGGATTCGCCAGACAATCGAACGGGCGATCATGAACCAAACCCG  480
Sbjct  421  ............................................................  480

Query  481  TACGATTCGCTTGCCGATTCACATTGTTAAAGAGCTGAACGTATACCTGCGCACCGCACG  540
Sbjct  481  ............................................................  540

Query  541  TGAGTTGTCGCATAAACTGGACCACGAACCGAGTGCGGAAGAAATTGCAGAGCAACTGGA  600
Sbjct  541  ............................................................  600

Query  601  TAAACCGGTTGATGACGTCAGCCGTATGCTTCGTCTCAACGAGCGCATTACCTCGGTAGA  660
Sbjct  601  .........................................--------...........  652

Query  661  CACCCCGCTGGGCGGTGATTCCGAAAAAGCGTTGCTGGACATCCTGGCCGATGAAAAAGA  720
Sbjct  653  ............................................................  712

Query  721  GAACGGTCCGGAAGACACCACGCAAGATGACGATATGAAACAGAGCATCGTCAAATGGTT  780
Sbjct  713  ............................................................  772

Query  781  GTTCGAACTGAACGCCAAACAGCGTGAAGTGCTGGCGCGCCGTTTCGGTCTGCTGGGATA  840
Sbjct  773  ............................................................  832

Query  841  TGAAGCTGCGACACTGGAAGATGTAGGCCGTGAAATCGGTCTTACGCGTGAACGTGTTCG  900
Sbjct  833  ............................................................  892

Query  901  TCAGATTCAGGTTGAAGGCCTGCGCCGTCTGCGCGAAATTCTGCAGACGCAGGGGCTGAA  960
Sbjct  893  ............................................................  952

Query  961  TATCGAAGCGCTGTTCCGCGAGTAA  985
Sbjct  953  .........................  977
```

B

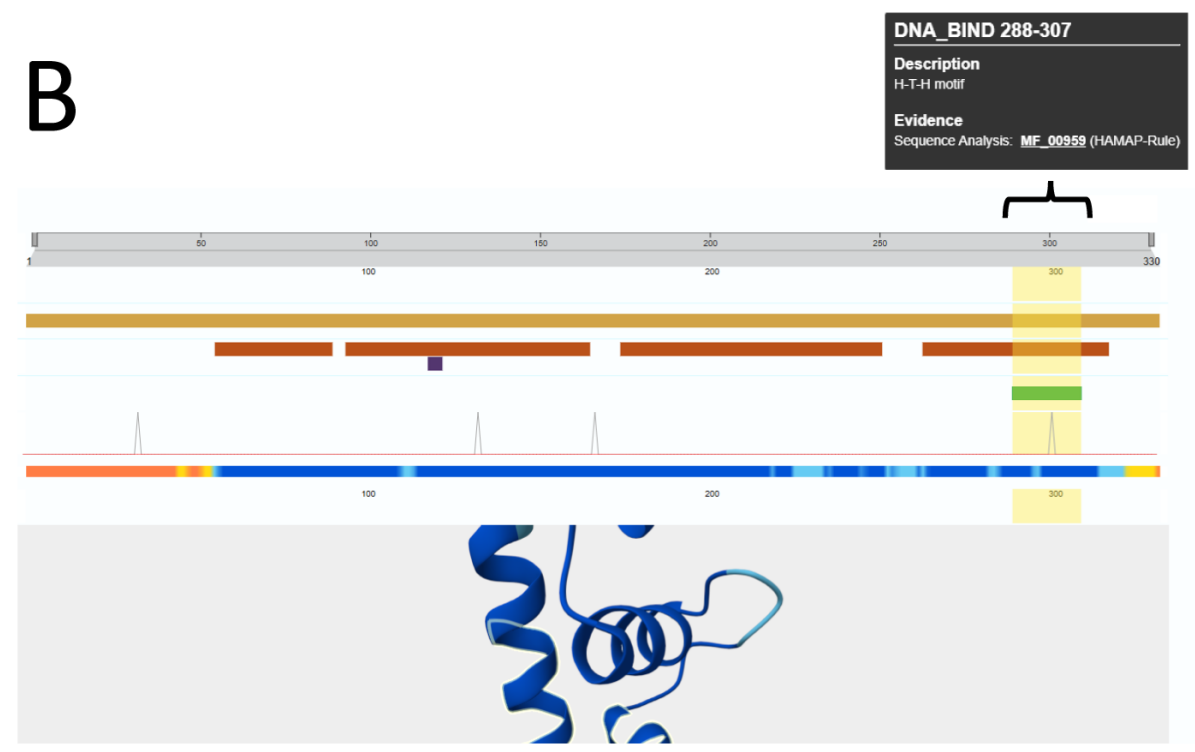

Figure S3. Serine sensitivity of *ridA* (LT2) is unaffected by compromising *rpoS*. Growth of WT(LT2) (DM9404), *ridA*(LT2) (DM3480), *rpoS*(LT2) (DM15340) and *rpoS ridA*(LT2) (DM15341) in glucose minimal medium with (A) no addition, (B) 5 mM serine, (C) 5 mM serine and 1 mM isoleucine, or (D) 5mM serine, 0.667 mM glycine, and 1 µM thiamine. Data points represent the mean of three biological replicates with error bars indicating standard deviation.

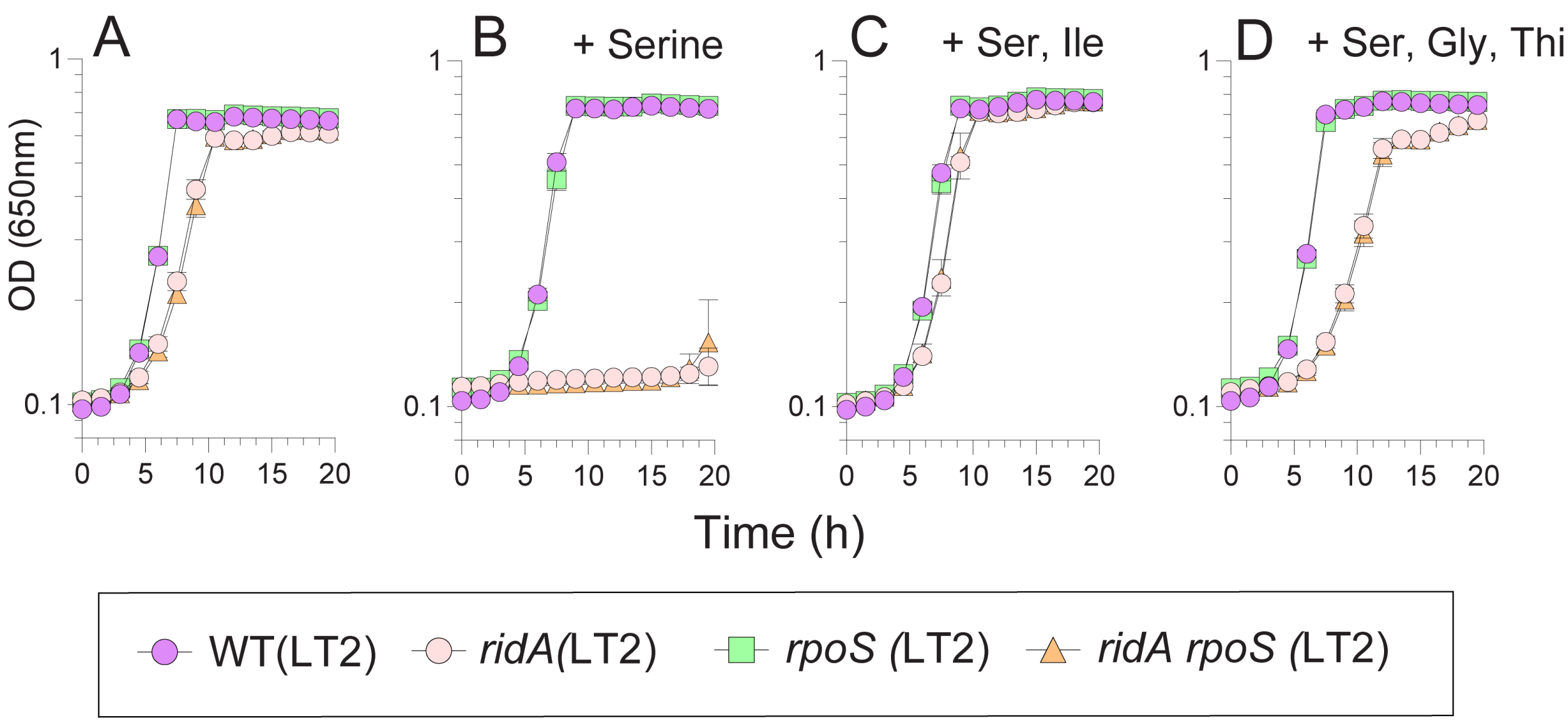

REFERENCES

Castilho, B. A., Olfson, P., & Casadaban, M. J. (1984). Plasmid insertion mutagenesis and lac gene fusion with mini-mu bacteriophage transposons. *Journal of Bacteriology*, *158*, 488–495.

Porwollik, S., Chu, W., Desai, P. T., & McClelland, M. (2024). A genome-wide collection of barcoded single-gene deletion mutants in Salmonella enterica serovar Typhimurium. *PLoS ONE*, *19*(3 March). https://doi.org/10.1371/journal.pone.0298419

Way, J. C., Davis, M. A., Morisato, D., Roberts, D. E., & Kleckner, N. (1984). New Tn10 derivatives for transposon mutagenesis and for construction of lacZ operon fusions by transposition. *Gene*, *32*, 369–379.
